# Clinical effect of vestibular rehabilitation on benign paroxysmal positional vertigo: A protocol for systematic review

**DOI:** 10.1097/MD.0000000000023906

**Published:** 2021-01-22

**Authors:** Wei Liu, Cheng-Li Pan, Xi-chun Wang, Shuang Sun

**Affiliations:** a Department of Physical Examination, Shenzhen People's Hospital, Shenzhen; b Department of Geriatric Neurology, Heilongjiang Provincial Hospital, Harbin, China.

**Keywords:** benign paroxysmal positional vertigo, effect, vestibular rehabilitation

## Abstract

**Background:**

This study will evaluate the clinical effect of vestibular rehabilitation (VR) on benign paroxysmal positional vertigo (BPPV).

**Methods:**

In this study, we will identify relevant trials on the topic published in MEDLINE, EBASE, Web of Science, Cochrane Library, Scopus, CINAHL, CBM, and CNKI from inception to the present. We will also search conference proceedings, thesis/dissertation, ongoing trials in clinical trial registry, and reference lists of included studies. Two researchers will independently carry out record selection, data extraction, and study quality assessment, respectively. Any disagreement will be arbitrated and solved with the help of a third researcher. If necessary, we will conduct random-effects meta-analysis to pool the effect estimates of included trials determined to be acceptable heterogeneity.

**Results:**

We will summarize the latest evidence to assess the effect of VR for the treatment of patients with BPPV.

**Conclusion:**

The findings of this study will help determine whether or not VR is effective in treating BPPV.

**OSF registration:**

osf.io/k83y5.

## 1 Introducton

Vertigo is a very common symptom and reason for primary clinic practice visit.^[1–3]^ It is reported that about 7.4% general adult population experience such disorder in their lifetime.^[4,5]^ This figure increased in the elderly with an obvious female preponderance.^[4–6]^ Of it, benign paroxysmal positional vertigo (BPPV) accounts for most common type of vertigo.^[7–10]^ It occurs in the inner ear because of the changes in position.^[9–12]^ Although standard treatment is applied to manage such disorder, its 1-year recurrence rate is about 20%, and its 4 to 5 year recurrence rate is between 40% and 50%.^[13–15]^ Thus, effective treatments are still urgent to help manage such issue.

Studies suggested that vestibular rehabilitation (VR) can be utilized for the treatment of patients with BPPV.^[16–28]^ However, there is still insufficient evidence-based medicine evidence to support this topic. Thus, this study will systematically and comprehensively investigate the effect of VR for the treatment of BPPV.

## 2 Methods

### 2.1 Study registration

This study protocol was registered on OSF (osf.io/k83y5). It follows the guidelines of the Preferred Reporting Items for Systematic Review and Meta-Analysis Protocols standards.^[29]^

### 2.2 Eligibility criteria for study selection

#### 2.2.1 Type of studies

This study will include all randomized controlled trials on the topic of VR in treating BPPV. We will exclude animal study, case report, case series, review, comment, and uncontrolled study.

#### 2.2.2 Type of participants

This study will include eligible patients of any age who were diagnosed as BPPV, regardless their race, gender, and educational and economic background.

#### 2.2.3 Type of interventions and controls

In the treatment group, all patients received VR as their management.

In the control group, there are no limitations to the comparators. However, we will exclude control treatments involved any forms of VR.

#### 2.2.4 Type of outcomes

Outcomes include annual recurrence rate, vertigo intensity, vertigo symptoms, vestibular disability, time to recovery, mean blood velocity (Vm), health-related quality of life, and adverse events.

### 2.3 Search strategy

To examine relevant trials on the topic, this study will search records in MEDLINE, EBASE, Web of Science, Cochrane Library, Scopus, CINAHL, CBM, and CNKI from the beginning to the present. We will not impose any limitation to the language and time of publication. The sample of detailed search strategy for MEDLINE is summarized in Table 1. We will also adapt similar search strategy to the other electronic databases. In addition, we will examine conference proceedings, thesis/dissertation, ongoing trials in clinical trial registry, and reference lists of included studies.

**Table 1:** Detailed search strategy for MEDLINE.

Number	Search terms
1	Benign paroxysmal positional vertigo
2	Vertigo
3	Dizziness
4	Postural balance
5	Sudden sensation
6	Spinning
7	Moving
8	Unsteadiness
9	Dizzy
10	Or 1–9
11	Vestibular rehabilitation
12	Physical therapy
13	Physical modality
14	Physical treatment
15	Physical management
16	Or 11–15
17	Randomized
18	Randomly
19	Random
20	Blind
21	Concealment
22	Allocation
23	Controlled trial
24	Clinical trial
25	Control
26	Comparator
27	Trial
28	Study
29	Or 17–28
30	10 and 16 and 29

### 2.4 Literature selection and data extraction

#### 2.4.1 Literature selection

Two researchers will independently scan all identified, and all duplicated citations will be removed. There are 2 stages in the citation selection. First, we will examine all titles/abstracts of all potential records to eliminate all irrelevant studies. Second, full text of the rest articles will be carefully read against all inclusion criteria. If any discrepancy occurs, we will invite a third researcher to resolve it through discussion and a final decision will be made after discussion. The results of study selection will be presented in a flow diagram.

#### 2.4.2 Data extraction

Two researchers will independently carry out data extraction using previously designed data collection template. The extracted information consists of title, authors, year of publication, country, patient characteristics, diagnostic criteria, inclusion and exclusion criteria, trial design, trial setting, sample size calculation, details of VR and controls, outcomes, follow-up information, dropouts, finding details, and any other relevant information. Any division will be resolved by another experienced researcher through discussion. If we identify any unclear or insufficient or missing data, we will contact primary authors to obtain it through email or fax. If we can not achieve that data, we will only analyze available data, and will discuss its potential impacts to the study findings.

### 2.5 Study quality assessment

Two researchers will independently appraise study quality of all included trials through 7 aspects using Cochrane Risk of Bias Tool. Each filed is further rated as low risk of bias, unclear risk of bias, and high risk of bias. Any division will be resolved by a third researcher via consultation.

### 2.6 Statistical analysis

This study will undertake statistical analysis using RevMan 5.3 software. All continuous outcomes will be estimated as mean difference and 95% confidence intervals. All dichotomous outcome will be calculated as risk ratio and 95% confidence intervals. Statistical heterogeneity across trials will be quantified using *I*^*2*^ test. *I*^*2*^ ≤50% means acceptable heterogeneity, while *I*^*2*^ > 50% suggests significant heterogeneity. If there is acceptable heterogeneity, and sufficient data is extracted on the same outcome, we will carry out a random-effects meta-analysis according to the sufficient similarity in study information, patient characteristics, and details of VR and controls. If there is remarkable heterogeneity, we will carry out subgroup analysis to explore its any potential cause. If meta-analysis can not be conducted, we will construct tables to narratively describe and summarize the study findings.

### 2.7 Subgroup analysis

This study will organize a subgroup analysis to explore any possible factors that may result in obvious heterogeneity.

### 2.8 Sensitivity analysis

This study will carry out sensitivity analysis to examine the robustness of study findings by removing low quality studies.

### 2.9 Reporting bias

If more than 10 randomized controlled trials are included in this study, we will detect its reporting bias using Egger regression test^[30,31]^ to check funnel plot asymmetry.

### 2.10 Ethics and dissemination

This study does not require ethical approval, since no individual data will be collected. We plan to disseminate this study on a peer-reviewed journal or via conference meeting.

## 3 Discussion

Previous studies suggested that VR can be utilized for the treatment of BPPV. However, there is still insufficient evidence to support this topic. With the rising number of clinical trials reporting the effect of VR for BPPV, it is very necessary to pool the outcome data from eligible trials to accurately appraise its treatment effect. We will search both electronic databases and other resources to avoid missing potential trials. Then, we will incorporate outcome data from different trials into analyses. The findings of this study may provide evidence to support the effect of VR for the treatment of BPPV. However, this study still has several drawbacks. First, there may be insufficient number of eligible trials. Second, the sample size of included trials may be small. Third, the methodological quality of included studies may be poor. Fourth, the statistical heterogeneity of pooled outcomes may be remarkable. All those limitations may affect the findings of this study.

## Author contributions

**Conceptualization:** Wei Liu, Xi-chun Wang, Shuang Sun.

**Data curation:** Wei Liu, Cheng-Li Pan.

**Formal analysis:** Wei Liu, Cheng-Li Pan, Xi-chun Wang, Shuang Sun.

**Investigation:** Shuang Sun.

**Methodology:** Wei Liu, Xi-chun Wang.

**Project administration:** Shuang Sun.

**Resources:** Wei Liu, Cheng-Li Pan.

**Software:** Cheng-Li Pan, Xi-chun Wang.

**Supervision:** Shuang Sun.

**Validation:** Wei Liu, Cheng-Li Pan, Xi-chun Wang, Shuang Sun.

**Visualization:** Xi-chun Wang, Shuang Sun.

**Writing – original draft:** Wei Liu, Cheng-Li Pan, Xi-chun Wang, Shuang Sun.

**Writing – review & editing:** Wei Liu, Cheng-Li Pan, Shuang Sun.
